# Adalimumab Biosimilar in Pediatric Inflammatory Bowel Disease: A Retrospective Study from the Sicilian Network for Inflammatory Bowel Disease (SN-IBD)

**DOI:** 10.3390/healthcare12030404

**Published:** 2024-02-04

**Authors:** Valeria Dipasquale, Salvatore Pellegrino, Marco Ventimiglia, Michele Citrano, Francesco Graziano, Maria Cappello, Anita Busacca, Ambrogio Orlando, Salvatore Accomando, Claudio Romano, Sicilian Network for Inflammatory Bowel Disease

**Affiliations:** 1 Pediatric Gastroenterology and Cystic Fibrosis Unit, Department of Human Pathology in Adulthood and Childhood “G. Barresi”, University Hospital “G. Martino”, 98100 Messina, Italy; 2 Directorate General of Medical Device and Pharmaceutical Service, Italian Ministry of Health, 00153 Rome, Italy; 3 Pediatric Unit, Villa Sofia Cervello Hospital, 90146 Palermo, Italy; 4 Gastroenterology and Hepatology Section, Department of Health Promotion, Mother and Child Care, Internal Medicine and Medical Specialties (PROMISE), University of Palermo, 90128 Palermo, Italy; 5 IBD Unit, Department of Medicine, Villa Sofia Cervello Hospital, 90146 Palermo, Italy; 6 Pediatric Section, Department of Health Promotion, Mother and Child Care, Internal Medicine and Medical Specialties (PROMISE), University of Palermo, 90128 Palermo, Italy

**Keywords:** ABP501, biosimilar, effectiveness, GP2017, pediatrics, safety

## Abstract

Background: The utilization of anti-tumor necrosis factor-α (anti-TNF-α) biosimilars in inflammatory bowel disease (IBD) is constantly increasing. However, pediatric data are limited. This study aimed to assess the effectiveness and safety of adalimumab biosimilar (ADL-BioS) in pediatric IBD patients. Methods: All consecutive pediatric IBD patients from the Sicilian Network for Inflammatory Bowel Disease cohort treated with ADL-BioS from 2019 to 2021 were recruited. Remission at weeks 14 and 52, treatment persistence, and adverse events were the endpoints of this study. Factors associated with clinical remission and treatment persistence were examined. Results: There were 41 patients in total. Nine (22%) patients were switched from the reference product to ADL-BioS. Two patients had multiple switches. Eleven months was the median follow-up period. Clinical remission was attained by 70.7% and 72.0% of patients on weeks 14 and 52, respectively. Four (9.8%) adverse events occurred (10.1/100 person-year). Treatment persistence was 85.4% at 1 and 2 years. Patients with a longer duration of disease had a higher probability of stopping their treatment (*p* = 0.036). Conclusions: This is the first real-world study that particularly addresses the use of ADL-BioS in pediatric IBD. With high rates of treatment persistence and a low frequency of non-serious side effects, ADL-BioS seems to be effective.

## 1. Introduction

Tumor necrosis factor-α (TNF-α) inhibitors were the first biologic drugs approved for the treatment of inflammatory bowel disease (IBD). Adalimumab (ADL) (Humira^®^) is a fully humanized IgG1 monoclonal antibody with a high affinity for TNF-α and was introduced as the second TNF-α inhibitor after infliximab (IFX) (Remicade^®^) [1]. The high cost of TNF-α inhibitors is one of their major drawbacks [2]. The product patent for ADL expired in Europe in October 2018, resulting in the development of a robust pipeline of low-cost ADL biosimilars (ADL-BioS) [3]. ABP 501 (Amgevita^®^) was the first ADL biosimilar to be approved in Europe in March 2017 after extensive, high-quality, randomized controlled studies in patients with rheumatoid arthritis and psoriasis demonstrated its overall clinical comparability to the originator [4,5]. So far, there are only a few observational studies on the use of ADL-BioS in adult patients with IBD, reporting similar safety and effectiveness profiles to those described for the ADL originator even after switching [6,7,8,9]. However, these studies are all limited to adult patients. To the best of our knowledge, this is the first real-life study specifically addressing the use of ADL-BioS in pediatric IBD patients. The main aim was to investigate the short- and medium-term effectiveness and safety of ADL-BioS. Patients were identified among the cohort of the Sicilian Network for Inflammatory Bowel Disease (SN-IBD), a web-based prospective registry that includes data on patients with IBD treated with biologics and followed up in one of the 16 centers licensed to prescribe biologics in Sicily (Italy).

## 2. Materials and Methods

This was a multicenter, retrospective, observational study conducted within the SN-IBD. All consecutive pediatric IBD patients diagnosed according to the Porto criteria [10] and treated with ADL-BioS were enrolled. In Sicily, the first ADL-BioS (ABP501) was available in clinical practice as of February 2019. Data from February 2019 to January 2021 were collected from the SN-IBD registry. The study end date was determined to be the date of the last clinical visit prior to the retrieval date. ADL-BioS was administered in accordance with the indications and dosing, which included the possibility of optimization at the discretion of the physician. Gender, age at diagnosis, disease location and behavior according to the Paris classification [11], first-degree familiarity, extraintestinal manifestations, surgery, and previous therapies were recorded. Data on ADL-BioS treatment included age at initiation, concomitant therapies, optimization, the need for adjunctive therapies, and treatment duration. The Pediatric Crohn’s Disease Activity Index (PCDAI) for CD and the Pediatric Ulcerative Colitis Activity Index (PUCAI) for UC, hemoglobin (Hb), the erythrocyte sedimentation rate (ESR), C-reactive protein (CRP), and fecal calprotectin at the baseline, 14 and 52 weeks, were collected. Adverse events were recorded.

### 2.1. Study Outcomes

ADL-BioS efficacy was evaluated on weeks 14 and 52. All patients who completed 52 weeks of treatment and all previous failures were included in the analysis of 52-week outcomes. Remission was defined as PCDAI or PUCAI < 10. A partial response was defined as a change of at least 20 points from the baseline. ADL-BioS failure was defined as a lack of clinical response at the end of the induction (primary failure) and the loss of efficacy after an initial response (secondary loss of response). Secondary outcomes were treatment persistence (defined as the time from drug initiation to the discontinuation of therapy) and the adverse event rate.

### 2.2. Statistical Analysis

Continuous variables were reported as medians with interquartile ranges (IQRs), and categorical variables were reported as frequency and percentage. The Mann–Whitney U-test and χ2 tests (or Fisher’s exact test if required) were used to compare continuous and categorical data, respectively. Paired comparisons between laboratory variables were performed using Wilcoxon’s tests. Univariable logistic regression analyses were used to find independent predictors of clinical response. Univariable Cox proportional hazard (PH) models were used to test the association between baseline factors and both clinical remission and treatment persistence. Due to a small number of observations, no multiple regression model (both logistic and Cox PH) was fitted. The Schoenfeld test was used to evaluate proportional hazard assumptions, and they were not violated. The logistic and Cox PH models were both fitted using Firth’s bias reduction method [12] to overcome the problem of data separation induced by small sample size and/or imbalanced or strongly predictive risk factors [13]. R version 4.1.1 (R Foundation for Statistical Computing, Vienna, Austria) was used for all statistical analyses [14]. The statistical significance level was set at 0.05.

## 3. Results

Forty-one pediatric patients on ADL-BioS from Sicilian IBD units were included (the total number of patients on ADL: n = 100): 39 (95.1%) with CD, 1 (2.4%) with UC, and 1 with IBD-U (2.4%). The patient with UC was started on ADL because of an intolerance to IFX. The baseline characteristics of all patients are summarized in Table 1.

The UC patient had extensive disease (hepatic flexure distally), while the IBD-U patient had only colonic involvement. All children started ADL-BioS at the standard dose (a 20 and 40 mg subcutaneous injection every 2 weeks for children under and over 40 kg, respectively, after the initial induction treatment). Nine (22%) patients were switched from the originator to ADL-BioS. The switch occurred when they were in remission. The biosimilars prescribed were ABP501 (Amgevita^®^, n = 38) and GP2017 (Hyrimoz^®^, n = 5). Due to local availability, two (4.9%) patients underwent multiple switches (>1 switch) between biosimilars (both from ABP501 to GP2017).

**Figure 1:**
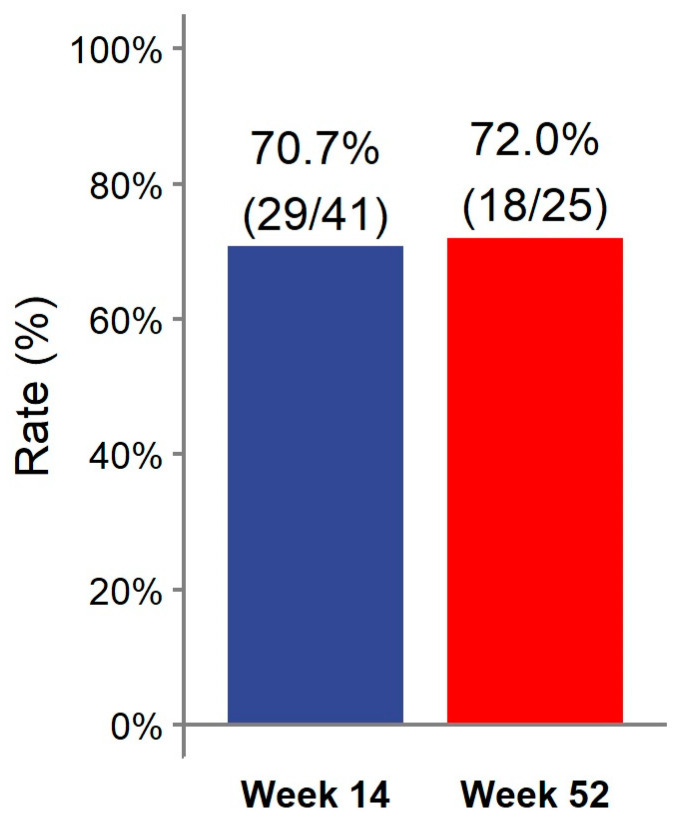
Clinical remission rate at weeks 14 and 52.

**Figure 2:**
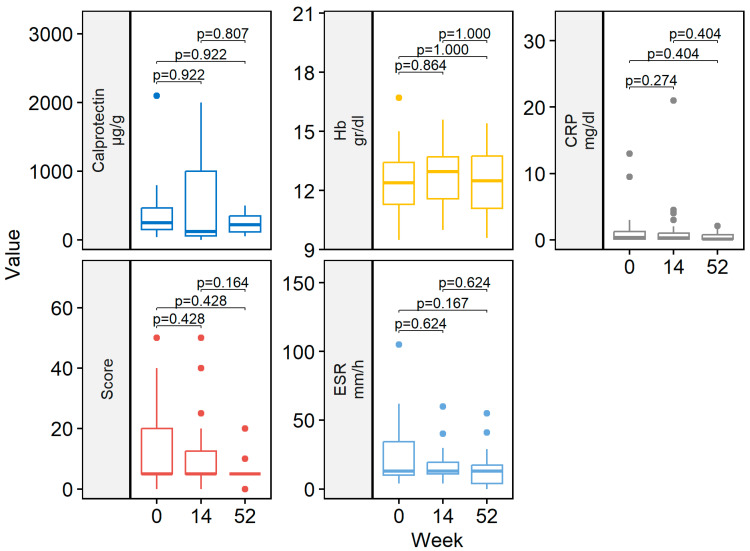
Boxplot of clinical scores and laboratory data by follow-up weeks (baseline, week 14 and 52). CRP, C-reactive protein; ESR, erythrocyte sedimentation rate; Hb, hemoglobin.

At week 14, 29/41 (70.7%) patients achieved clinical remission (Figure 1). Eight (19.5%) patients had a partial response. Two patients discontinued ADL-BioS before reaching week 14 due to primary failure (n = 1) and adverse events (n = 1). One (2.4%) patient only required dose escalation during the first 14 weeks of treatment. Clinical scores and laboratory parameters at weeks 14 and 52 are represented in Table 2. At week 14, no differences in the clinical score, CRP, ESR, Hb, or fecal calprotectin were detected compared to the baseline (Figure 2).

Almost all patients treated with ADL-BioS (39/41; 95.1%) continued treatment after induction. Twenty-five (61%) patients continued treatment through week 52. The clinical remission analysis at 52 weeks, calculated in patients who reached 52 weeks of treatment and all previous failures, considered non-responders and showed the following results: 18/25 (72.0%) patients were in remission (Figure 1) (notably, 16 of these were already in remission on week 14 and maintained in remission at week 52); two (8.0%) children had a partial response. Seven (17.1%) patients required treatment escalation during maintenance therapy: five children had the dose increased, and two children had an interval between doses, resulting in a decrease. Only two (4.9%) patients required steroids at any time during the maintenance phase. Clinical scores and laboratory parameters (CRP, ESR, Hb, fecal calprotectin) were similar between week 52 and both the end of induction (week 14) and the baseline (Figure 2).

### 3.1. Predictive Factors Associated with Remission

Remission at week 14 was reported more frequently in patients with shorter disease duration (OR 0.72, 95% CI 0.53–0.95, *p* = 0.029). Gender, first-degree familiarity, the type of IBD, age at onset of BioS, age at diagnosis, disease location, perianal involvement, extraintestinal manifestations, prior surgery, being naïve to anti-TNF-*α*, switching from the originator, and laboratory data (CRP, ESR, Hb, fecal calprotectin) were not associated with achieving remission at the end of induction. A significant association between disease duration and remission (the shorter the disease duration, the higher the remission rate) was found on week 52 as well (OR 0.65, 95% CI 0.42–0.92, *p* = 0.027). In addition, at week 52, patients on ADL-BioS who achieved remission did not switch from the originator (OR 0.08, 95% CI 0.01–0.55, *p* = 0.016) and had lower CRP values at the baseline (OR 0.23, 95% CI 0.05–0.73, *p* = 0.03), compared to those who did not achieve remission. Gender, first-degree familiarity, the type of IBD, age at starting BioS, age at diagnosis, disease location, perianal involvement, extraintestinal manifestations, previous surgery, being naïve to anti-TNF-*α*, and other laboratory data (ESR, Hb, fecal calprotectin) were not associated with achieving remission at week 52.

### 3.2. Duration of ADL-BioS Treatment

**Figure 3:**
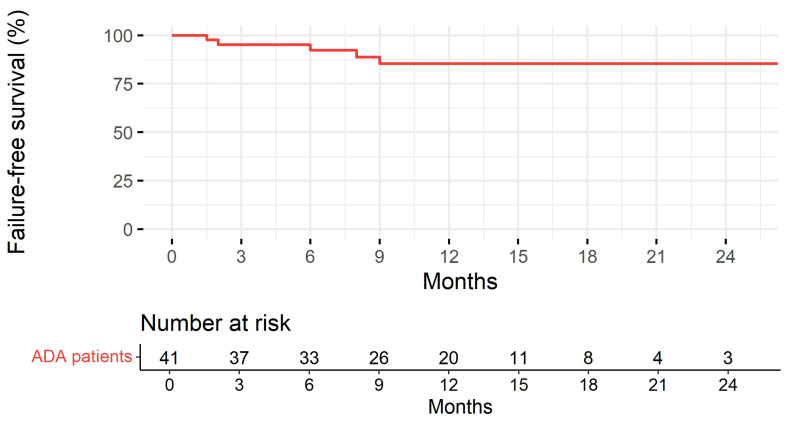
Treatment persistence with adalimumab biosimilar estimated according to the Kaplan–Meier method and table with the number of subjects at risk.

Patients were followed up for a median of 11 months (IQR: 7.00–15.0). Treatment persistence at 1 and 2 years is represented in Figure 3. Disease duration was significantly associated with the risk of treatment discontinuation (the longer the disease duration, the higher the risk of treatment discontinuation) in the univariable Cox regression model analysis (HR 1.38, 95% CI 1.02–1.87, *p* = 0.036). No association was found with gender, first-degree familiarity, the type of IBD, age at starting BioS, age at diagnosis, disease location, perianal disease, extraintestinal manifestations, prior surgery, being naïve to anti-TNF-*α*, switching from the originator, and laboratory parameters (CRP, ESR, Hb, fecal calprotectin).

### 3.3. Safety

A total of four (9.8%) adverse events were registered, with an overall incidence of 10.1/100 person-year. They were represented by psoriasiform dermatitis (n = 2), acute injection reactions (n = 1), and infections (urinary tract infections, vulvar and oral abscesses, n = 1). Adverse events led to drug discontinuation in two cases. Notably, 1 out of every 2 patients who switched more than one biosimilar experienced a rash requiring treatment withdrawal.

## 4. Discussion

Biological agents with strong anti-inflammatory action, such as anti-TNF agents, have modified the IBD treatment scheme and goals. A number of biological agents are being studied in pediatric populations. Currently, only two biological agents, IFX and ADL, are licensed for use in children and adolescents. There are risks and burdens associated with allowing mucosal repair when using these two biological agents in pediatric IBD. ADL is a 100% human anti-TNF monoclonal antibody that specifically binds to TNF-α. Following the induction phase, children weighing less than or more than 40 kg are usually given a subcutaneous injection of 20 or 40 mg every two weeks. ADL can be used to treat moderately to severely active CD in pediatric patients (aged 6 years and older) who experience an inadequate response to or are intolerant of conventional therapy, such as primary nutritional therapy, a corticosteroid, and/or an immunomodulator, or who have contraindications for these treatments. ADL was first licensed for pediatric CD in 2012 and has been in use for over 9 years, gathering a substantial body of empirical data [1,15,16]. ADL has not yet been approved to treat pediatric UC. The current guidelines state that in cases of loss of response or intolerance to IFX, ADL should be considered [17]. To the best of our knowledge, this is the first study specifically evaluating the effectiveness and safety of ADL-BioS in pediatric CD and UC patients. High clinical remission rates at both weeks 14 and 52 (70.7% and 72.0%, respectively) were noted, with high rates of treatment persistence at 1 and 2 years. Some patients (one during the induction and seven from week 14 up to week 52) required treatment escalation. No significant improvements in clinical scores and laboratory values were reported during the study period; however, both median scores and inflammatory markers were within their normal ranges for age, even at the baseline, making improvement across study time points unlikely. Interestingly, it was found that disease duration before starting the biosimilar was inversely associated with clinical remission at weeks 14 and 52 and with treatment persistence at 1 and 2 years. This result is of clinical relevance as it confirms the relevant role of early and timely treatment for better outcomes and treatment response [18]. On week 52, an inverse association was found between CRP at the baseline and clinical remission. This could be explained by the fact that a higher CRP at the baseline indicates more severe inflammation and disease, which, on the one hand, represents an indication to start biosimilar therapy and, on the other hand, is associated with a worse response to treatment or a lower sustained response to treatment over time.

This study included patients naïve to anti-TNFs, those who switched from an ADL originator to an ADL-BioS, and those who switched between biosimilars multiple times. A previous SN-IBD retrospective study found that switching from an originator to ABP501 was safe and effective in adult IBD patients [9]. Adult IBD patients who were treated with an ADA biosimilar ABP 501 between February 2019 and February 2020 (n = 599) among the SN-IBD cohort were enrolled. The patients were divided into three groups for study purposes: group A was newly exposed to ADA and anti-tumor necrosis factors; group B was previously exposed to anti-tumor necrosis factors and was naïve to ADA; and group C was switched from the ADA originator to ABP 501. Significant adverse events occurred in thirty-six patients (6.4%; incidence rate: 8.9 per 100 person-year). Patients in group B compared with group C had a higher incidence rate of serious adverse events (16.4 vs. 4.8 per 100 person-year; incidence rate ratio = 3.42; *p* = 0.041), and patients in group A compared with group C (17.4 vs. 4.8 per 100 PY; incidence rate ratio = 3.61; *p* < 0.001). After 12 weeks, 188 (85.8%) of the ADA-naïve patients (group A + B) showed a clinical response, with 165 (75.3%) of them achieving steroid-free remission. Patients in group C had higher treatment persistence estimates than those in groups A and B (*p* < 0.001) [9]. In the present study, subgroup analyses were not performed because the sample size was too small. However, switching from the originator to ADL-BioS was found to be inversely related to clinical remission at week 52. Switching to biosimilars may lead to an increased risk of immunogenicity. Immunogenicity, such as the development of antibodies to IFX (ATIs), is associated with loss of response, acute injection reactions, and delayed hypersensitivity reactions and can differ between different biologics due to the differences in their formulation, purity, or packaging [19,20]. However, this was not investigated in this study.

The rate (9.8%) and characteristics of adverse events were consistent with those described for ADL-BioS in adults [9], as well as for the ADL originator [21], with no unexpected serious outcomes.

This study has some limitations. First, this study was retrospective, so the included cases and clinical management approaches acted as confounding variables. Second, IBD children on the ADL originator were not included in the study to compare its effectiveness and safety to those of ADL-BioS. However, many studies have already been published on the efficacy and safety profile of the ADL originator for this group of patients [15,16]. The aim of the present study was to provide real-life data on the use of ADL-BioS in pediatric CD and UC patients in order to address possible concerns about its use. Third, no data were available on ADL trough levels or antidrug antibodies, making it impossible to assess the pharmacokinetic profile or whether, for example, children who switched ≥1 once had increased immunogenicity. Fourth, the study’s small sample size precludes drawing firm conclusions (especially for patients who were switched from the originator, n = 9). A larger sample size would allow for more powerful statistical analysis and the use of more complex statistical tools (e.g., multiple regression analyses). Finally, endoscopic data and fecal calprotectin levels were incomplete, limiting the ability to assess mucosal healing. A strength of this study is its specific focus on the use of ADL-BioS in pediatric IBD patients. In addition, patients were drawn from a regional-based patient registry, with all participating centers following the same protocols of treatment, so the follow-up data were homogeneous.

## 5. Conclusions

To the best of our knowledge, this is the first study to provide specific insights into the effectiveness and safety of ADL-BioS in pediatric IBD patients, with high rates of clinical remission rates and treatment persistence over time and a low incidence of mild adverse events. More research is needed to validate these findings and to explore the pharmacokinetics and immunogenicity of these biosimilars in pediatric IBD.

## Figures and Tables

**Table 1:** Baseline characteristics of patients.

Variable	Total (n = 41)
Gender, n (%)	
F	18 (43.9)
M	23 (56.1)
First-degree familiarity, n (%)	
No	37 (90.2)
Yes	4 (9.8)
Age at diagnosis (years), median [IQR]	13.6 [11.3;15.8]
Age at diagnosis for CD (years), n (%) *^,^**	
A1a: <10	9 (25.7)
A1b: 10–17	22 (62.9)
A2: 17–40	4 (11.4)
Disease location for CD, n (%) **	
L1: Distal 1/3 ileal + limited cecal disease	8 (20.5)
L2: Colonic	2 (5.1)
L3: Ileocolonic	22 (56.4)
L4: Upper disease	1 (2.6)
L3L4: Ileocolonic + upper disease	5 (12.8)
L1L4: Distal 1/3 ileal + limited cecal disease + upper disease	1 (2.6)
Disease behavior for CD, n (%) *^,^**	
Nonstricturing nonpenetrating	18 (60)
Stricturing	11 (36.7)
Penetrating	1 (3.3)
Perianal disease for CD, n (%) **	4 (10.2)
Growth delay for CD, n (%) *^,^**	6 (35.3)
Age at ADL-BioS start (years), median [IQR]	16.0 [14.0;17.6]
Disease duration (years), median [IQR]	0.62 [0.19;2.55]
Extraintestinal manifestation, n (%)	
No	35 (85.4)
Yes	6 (14.6)
Arthritis	4 (9.7)
Erythema nodosum	2 (4.8)
Previous surgery, n (%)	
No	35 (85.4)
Yes	6 (14.6)
Previous treatment, n (%)	
Steroids	25 (61.0)
Azathioprine	14 (34.1)
Mesalamine	9 (22.0)
Metronidazole	8 (19.5)
Infliximab	4 (9.8)
Exclusive enteral nutrition	1 (2.4)
Anti-TNF-α naïve, n (%)	
No	4 (9.8)
Yes	37 (90.2)
Switch from the originator, n (%)	
No	32 (78.0)
Yes	9 (22.0)
Scores and laboratory data (baseline), median [IQR] *	
PCDAI/PUCAI	5.00 [5.00;20.0]
CRP (mg/dL)	0.34 [0.11;1.27]
ESR (mm/h)	13.0 [10.0;34.2]
Hb (gr/dL)	12.4 [11.3;13.4]
Fecal calprotectin (µg/g)	250 [150;462]
Concomitant drugs, n (%) ***	
Steroids	11 (26.8)
Mesalamine	5 (12.2)
Azathioprine	3 (7.3)
Antibiotics (metronidazole, ciprofloxacin)	2 (4.9)

Abbreviations: ADL-BioS, adalimumab biosimilar; IQR, interquartile range; PCDAI, Pediatric Crohn’s Disease Activity Index; PUCAI, Pediatric Ulcerative Colitis Activity Index; CRP, C-reactive protein; ESR, erythrocyte sedimentation rate; Hb, hemoglobin. * Missing data. ** According to the Paris classification [11]. *** At induction.

**Table 2:** Clinical scores and laboratory data at weeks 14 and 52.

Scores and Laboratory Data	Total (n = 41)
Time at 14 weeks, median [IQR] *	
PCDAI/PUCAI	5.00 [5.00;12.5]
CRP (mg/dL)	0.28 [0.11;1.00]
ESR (mm/h)	13.0 [11.0;19.5]
Hb (gr%)	12.9 [11.6;13.7]
Fecal calprotectin (µg/g)	120 [60.0;1000]
Time at 52 weeks, median [IQR] *	
PCDAI/PUCAI	5.00 [5.00;5.00]
CRP (mg/dL)	0.11 [0.11;0.75]
ESR (mm/h)	13.0 [4.0;17.5]
Hb (gr%)	12.5 [11.1;13.8]
Fecal calprotectin (µg/g)	220 [118;350]

Abbreviations: IQR: interquartile range; PCDAI: Pediatric Crohn’s Disease Activity Index; PUCAI: Pediatric Ulcerative Colitis Activity Index; CRP: C-reactive protein; ESR: erythrocyte sedimentation rate; Hb: hemoglobin. * Missing data.

## Data Availability

The data presented in this study are available on request from the corresponding author.
